# Internet access and partnership formation in the United States

**DOI:** 10.1080/00324728.2021.1999485

**Published:** 2021-11-23

**Authors:** Maria Sironi, Ridhi Kashyap

**Affiliations:** 1 University College London; 2 University of Oxford

**Keywords:** Internet access, technology, union formation, life course, NLSY97, CPS

## Abstract

The Internet has fundamentally altered how we communicate and access information and who we can interact with. However, the implications of Internet access for partnership formation are theoretically ambiguous. We examine their association using data from the National Longitudinal Survey of Youth (NLSY97) and Current Population Survey (CPS) in the United States. We find that the relationship between Internet access and partnership states (in the NLSY97) or partnership status (in the CPS) is age-dependent. While negative at the youngest adult ages, the association becomes positive as individuals reach their mid- to late 20s, for both same-sex and different-sex partnerships. The results suggest that Internet access is positively associated with union formation when individuals enter the stage in the young adult life course when they feel ready to commit to a long-term partnership. Our study contributes to a growing literature that highlights the implications of digital technologies for demographic processes.

Supplementary material for this article is available at: https://doi.org/10.1080/00324728.2021.1999485

## Introduction

The rapid diffusion of the Internet has been one of the most significant social phenomena of the new millennium. In the United States (US), residential high-speed Internet usage grew from 5 to 74 per cent between 2000 and 2009 (Dettling 2017). By 2015, the proportion of American adults who were Internet users was 86 per cent (Pew Research Centre 2019). The expansion of the technology has had wide-ranging social and economic implications and has generated a seismic shift in how people access information and communicate with each other (DiMaggio et al. 2001). In contrast to other communication technologies such as the telephone, which largely improved communication within existing networks, the Internet has broadened the scope for social interaction by enabling new possibilities for individuals to find and meet people outside their existing social networks.

One domain in which the opportunities afforded by the Internet—for individuals to communicate more freely, access a wider range of information, and reach outside their network—has been particularly significant is in finding new romantic partners. The Internet has been described as ‘the new social intermediary in the search for mates’ (Rosenfeld and Thomas 2012). Drawing on a nationally representative survey of 4,000 adults in the US who were already in relationships, Rosenfeld and Thomas (2012) found that 3.9 per cent of those that met in 1994–98 reported having first met online, increasing to 20 per cent of those who met in 2004–06. For couples who met in 2017, nearly 40 per cent met first online. By 2013 the Internet had become the most common way of meeting a partner for heterosexual couples in the US, surpassing meeting through friends (Rosenfeld et al. 2019). Hybrid online–offline modes of meeting partners have also grown at a steady rate, as social networking websites have made reconnecting after offline introductions easier (Thomas 2020).

Although Rosenfeld and colleagues highlighted the significant role of the Internet as an intermediary for those that were coupled (Rosenfeld and Thomas 2012; Rosenfeld et al. 2019), the relationship between Internet access and the propensity to partner or transition to committed unions such as marriage is theoretically ambiguous. On one hand, the greater amount of information on prospective partners and easier communication opportunities that the Internet affords may predict a faster transition to a committed partnership or marriage (Rosenfield, 2017) and better quality, more stable matches (Hitsch et al. 2010; Cacioppo et al. 2013). For example, Internet dating platforms and social networking sites allow users to access a wider pool of partners and to sort and search for those that meet user-defined criteria and shared interests. The Internet also affords easier opportunities for communication and quicker reconnection for those who first meet offline. Together, all these features could result in more efficient matching. Conversely, those sceptical of the impact of the Internet have argued that the abundance of choice of potential mates afforded by the Internet may lead to ‘choice overload’ and the inability to commit to a partnership (Yang and Chiou 2010; Turkle 2016). The choice overload argument posits that the increased choice set of potential partners provided by the Internet may make it harder to determine which is the best choice and consequently makes individuals less likely to commit to any choice. Increased time spent on the Internet on activities unrelated to partner search or communication with prospective partners may also crowd out time spent looking for a partner (Billari et al. 2019).

Despite the theoretical speculation about the relationship between Internet access and propensity to form a partnership or to marry, the empirical literature on the question has been relatively limited. An exception here is Bellou (2015), who exploited variation in the timing of broadband diffusion at the county level in the US to examine its impact on aggregate-level marriage rates. Bellou (2015) found the effect of broadband diffusion on marriage rates to be positive. While Rosenfeld and Thomas (2012) and Rosenfeld et al. (2019) found that an increasing fraction of couples were likely to meet online, Bellou’s findings when combined with those of Rosenfeld and colleagues suggest that the Internet may not just be displacing offline modes of meeting partners (e.g. through friends) but also generating altogether new matches that might not have otherwise occurred. In enabling new types of matches, the role of the Internet is likely to be especially salient for couples who might have had limited opportunity to meet and interact. Rosenfeld and Thomas (2012) have described this in terms of those seeking matches in ‘thin markets’, for example lesbian and gay individuals. Yet another possibility is that the implications of technology may vary across stages of the young adult life course. Technology may function as an enabling force for those looking to settle down, when age norms surrounding partnership or marriage become stronger, but may delay this transition for others who are not ready for this commitment.

Our study focuses on the association between Internet access and partnership formation at the individual level, examining its role for both different-sex and same-sex partnerships in the US. We analyse this relationship across two nationally representative data sources: the National Longitudinal Study of Youth (NLSY97) and the Current Population Survey (CPS). While the motivation of our paper is closely aligned with that of Bellou (2015), we use different data sources and analyse partnerships among different types of couple, including all co-residential unions rather than marriage only. In contrast to Bellou, who used aggregate-level data to study the relationship between Internet diffusion and marriage rates, we use individual-level data to analyse the association between Internet access and recurrent partnership *states* (from the NLSY97) and between Internet access and partnership *status* (from the CPS), controlling for a wide range of demographic and socio-economic confounders. An important contribution of our work compared with previous studies is that we examine how Internet access is associated with propensities to form different- and same-sex partnerships, including both marriages or cohabiting unions and at different stages of the (young) adult life course. By drawing on both cohort (longitudinal) data from the NLSY97 and period (cross-sectional) data from the CPS, we are able to assess whether Internet access is associated with individuals’ partnership outcomes only among a specific young adult cohort (as in the NLSY97) or whether the age-specific associations we find in our longitudinal analysis apply also to different cohorts (aged 15–50) over a longer period of Internet diffusion from 1997 onwards (in the CPS analyses). Although we account for a wide range of demographic and socio-economic confounders and perform several robustness analyses, we recognize that the potential endogeneity of Internet access prevents us from making causal claims. This limitation also stems in large part from the narrow range of digital technology use measures in nationally representative social and demographic surveys, a point we reflect on in our conclusions.

This paper proceeds as follows: in the next section, we consider mechanisms through which Internet access can be related to partnership formation and develop hypotheses for their relationship. We then present the data, methods, and results for each data source, and follow this with an overall discussion of our findings.

## Theoretical background and hypotheses

There are different ways in which the Internet could be associated with partnership formation. Although a growing literature has examined online dating (e.g. Potârcă and Mills 2015; Potârcă et al. 2015; Whyte and Torgler 2017; Rosenfeld 2018; Schwartz and Velotta 2018; Bruch and Newman 2019), the role of Internet access for partnership formation is likely more far-reaching and extends beyond enabling access to online dating websites and apps. Access to the Internet can affect partnership formation by: (1) expanding access to a wider pool of potential partners; (2) providing more information about and opportunity to reconnect with potential partners; and (3) facilitating easier and more distinctive forms of communication than those afforded by older technologies such as the telephone. While Internet access may affect partnership formation through these different channels, the direction of this relationship is a priori unclear. We first consider how these mechanisms may result in a positive link between Internet access and partnership formation, and then describe how this link may be negative. We conclude this section by further developing hypotheses about how these positive and negative associations may vary at different stages of the young adult life course.

Different digital platforms can facilitate the dating and partner search process by expanding access to a wider pool of potential partners, and venues for meeting online have become more numerous and diverse over the years (Cacioppo et al. 2013). Through Internet dating websites and mobile apps, social networking platforms, and shared discussion groups and posting boards, the Internet provides the chance to meet new people and draw on a wider network of individuals than those encountered in daily routines and through existing family and friend networks (Rosenfeld and Thomas 2012; Cacioppo et al. 2013; Bellou 2015; Rosenfield 2017). In this sense, Internet access can be seen as simplifying the search for a partner through the ability to screen a larger pool of potential partners. While other modes of online meeting were more popular prior to the mid-2000s, online dating sites and apps experienced rapid growth after that period, especially as smartphones took off. The uptake of Internet dating apps and websites in the US has been remarkable: a nationally representative Pew survey from 2019 found that nearly half (48 per cent) of young adults (aged 18–29) and 38 per cent of those aged 30–49 had used online dating apps (Pew Research Center 2020). The role of Internet access in simplifying partner search through a widened partner pool may be even more pronounced for subgroups within thin markets for potential partners, for example lesbian, gay, or bisexual individuals (Rosenfeld and Thomas 2012; Thomas 2020) or those with personality traits that might disadvantage their ability to meet in other ways (Danielsbacka et al. 2019). For example, even though more extroverted individuals have been shown to be more likely to use online dating and social media platforms (Valkenburg and Peter, 2007; Correa et al. 2010), Danielsbacka et al. (2019) found in a study using German data that individuals with less extroverted personality traits were more likely to have met their partner online.

In addition to a widened potential partner pool, online dating and social networking sites afford the opportunity to collect a lot of information and conduct a targeted search for prospective partners relatively quickly. Social networking sites and Internet search engines may also make reconnecting with those who individuals first meet offline (e.g. at a party) or have met in past social situations (e.g. in school) easier. This improved efficiency of the partner search process could hasten the process of partnership formation and increase the propensity to partner.

The immediacy and anonymity of communication afforded by the Internet could also predict a faster transition to partnership. Experimental evidence has suggested that anonymous online meetings promote greater self-disclosure and liking than face-to-face meetings (Bargh et al. 2002): McKenna et al. (2002) found that those who were able to disclose their inner selves over the Internet were also able to transition those relationships to real-life, face-to-face relationships. Even for those who first meet offline, the greater immediacy, connectivity, and privacy afforded by Internet-mediated communication could help with forging intimate bonds faster than in the absence of these technologies. Online communication through texting, chat, email, and social media—often conducted via smartphones—enables frequent and fast interactions, as well as both synchronous and asynchronous forms of communication that older communication technologies, such as the landline, could not. Communication is also more direct and personalized through the Internet, without the need to encounter any intermediaries. While calling a prospective romantic interest in the era of landlines might have meant calling and having to talk first with their family members, communication in a digital era means unrestricted, unmediated, and immediate access to a person of interest. For minority communities, such as lesbian, gay, or bisexual (LGB) individuals, who might face greater stigma or resistance towards their romantic interests, this effect is likely to be especially pronounced. Furthermore, for such individuals, access to online forums, groups, and communities may also act as a medium for both recognizing and validating their desires. In this way, the role of the Internet in partnership formation may be even stronger for LGB individuals, through both the search and information-seeking mechanisms.

While improved access to prospective partners, more information about them, and easier communication with them through the Internet may reduce the time and costs of partner search, the wider pool of partners provided by the Internet could also make the partner search process longer and imply a postponement in partnership formation. This negative relationship between Internet access and partnership formation can be understood from the perspectives of both search theory (Bellou 2015) and choice overload theory (Yang and Chiou 2010; Turkle 2016; Rosenfield 2017). Search theory posits that as the probabilities of receiving offers rises, so does the desired reservation quality. Applied to the partner search process, this implies that increased access to potential partners—and the likelihood of receiving offers from them—will lead to a higher set of expectations about the desired partner, which will in turn reduce the probability of transitioning to a partnership. From the perspective of choice overload theory, access to a larger pool of potential partners creates an abundance of choice that may make it harder to ‘settle down’ in the face of potentially unlimited possibilities to meet other (new) romantic partners. Too much choice, from this perspective, can be demotivating because individuals find it harder to determine which is the best choice, and having access to multiple options leads individuals to be less sure of their options and less likely to make any choice. In the context of initial formative interactions between strangers that are mediated online, studies suggest that individuals may over-interpret social cues, and this form of communication also lacks some of the experiential richness of face-to-face interaction (Finkel et al. 2012).

Another mechanism through which the Internet could negatively affect partnership formation is by crowding out the time spent looking for a partner (Billari et al., 2019). Early studies on whether the Internet displaced social activities found that greater time spent on the Internet at home, especially at weekends, negatively impacted on face-to-face interactions (Nie and Hillygus, 2002). Subsequent research, however, has argued that Internet use has not displaced offline social activities (Robinson and Martin 2010; Robinson 2011). While evidence on the effect of the technology on offline socializing may be mixed, it is nevertheless possible that with the diversification of opportunities for leisure, entertainment, or work that the Internet affords, these online activities may displace time spent online on activities linked to seeking a partner.

The discussion thus far suggests the hypothesis that the net effect of Internet access on partnership formation could go in either direction. Whether the relationship is positive or negative, however, could plausibly vary by age or stage of the life course: a dimension that has received limited discussion in the existing literature. Access to the Internet at younger ages may enable individuals to tap into a wider pool of romantic partners, benefit from ready availability of and easy communication with prospective partners, and potentially expand their dating opportunities. This may initially delay the transition to a long-term partnership. Alternatively, the purposes for which the Internet is used may vary at different ages. While at younger ages the Internet may be used mainly for purposes other than dating or the pursuit of romantic partnerships, at older ages individuals may begin to use the Internet more for partner search as their desire to partner (settle down) becomes stronger. This stage in the transition to adulthood—that follows the ‘emerging adulthood’ phase (Arnett 2000, 2014; Shanahan 2000; Schoen et al. 2007)—coincides with educational and employment transitions, and is a period in which an individual’s social network expands and their desire to live independently with a partner may also increase. Furthermore, the life-course approach emphasizes the importance of age norms for life-course decisions, and the desire to settle down with a partner may also emerge due to social norms about the appropriate timing for partnership formation as a life-course event (Billari and Liefbroer 2007). As young adults enter this stage, Internet access may facilitate the partnering process by providing targeted search and information about prospective partners, as well as easier modes of communication. These benefits can accrue not only to those who meet partners online, but also to those who first meet offline but use the Internet to communicate and learn more about their partner (e.g. through social networking sites). This discussion leads to the following hypothesis: at younger ages, we are likely to find that Internet access is negatively associated with the propensity to partner, that is, Internet access will be linked with partnership postponement. However, the improved search, efficiency, and communication afforded by the Internet may result in a positive association between Internet access and partnership formation at older ages.

Even as Internet access has proliferated in high-income countries (e.g. the US) over the twenty-first century, ‘digital divides’ in access to the Internet continue to exist across socio-demographic groups, with older, less educated, rural, and low-income individuals, as well as Black Americans, less likely to be Internet users (Warf 2013; Büchi et al. 2016). Existing research suggests, however, that several of these socio-demographic differences linked to social class, education, race, and urban/rural residence are also directly associated with the probability of partnership formation (Schwartz 2013; Tillman et al. 2019). For example, being from a higher social class not only increases the probability of achieving higher education, holding a better job, and obtaining a higher income in adulthood (all of which in turn affect Internet access) but in the US context also has a positive influence on the probability of forming a co-residential union (Sassler and Miller 2017; Tillman et al. 2019). Living in an urban area provides access to larger partner markets and wider social networks, which could affect the probability of partnership formation. An analysis of the relationship between Internet access and partnership thus needs to control for these potential confounders, which may independently affect partnership formation, an issue that we turn to with our empirical analysis.

## Empirical longitudinal analysis using the National Longitudinal Survey of Youth 1997

### Data and methods

The first set of analyses we present was conducted using the NLSY97. This data set includes a representative sample of young adults in the US, who were born between 1980 and 1984. They were interviewed for the first time in 1997 (when aged 12–17) and then every year after that until 2011. The NLSY97 collects data on socio-demographic characteristics, school and employment history, and partnership history (i.e. marriage and cohabitation, as information on dating history is either incomplete or limited). Importantly for our analysis, since 2003 the survey has included questions about Internet access. In particular, from 2003 to 2011, respondents were asked if they had access to the Internet, and from 2003 to 2008 they were asked from where they could access it (e.g. home, school, work, library, etc.). As the Internet access variable changed after 2011 to one on frequency of use, we limit our analysis to end in 2011 and exclude further waves from 2013, 2015, and 2017. Our key independent variable is based on the question *Do you currently have access to the Internet?*, and it is coded as ‘1’ if the answer is ‘yes’, and ‘0’ otherwise. We recognize that this measure of Internet access is clearly limited by not being able to capture *how* the Internet is used by the survey respondents. Nevertheless, an advantage of a general Internet access measure is that it covers broader forms of use, potentially capturing different mechanisms linked to access to information, networks, and communication afforded by the technology, as discussed in the previous section. Using a different data set, Thomas (2020) found that those with Internet access at home were more likely to have met their partner online, which suggests that this variable likely captures the use of the Internet for partner search.

Our main outcome of interest is partnership formation, including non-resident unions, cohabitation and marriage, and we are also interested in the sex of the partner to distinguish between different-sex and same-sex relationships. To study partnership transitions, we use both the co-residential household roster and the non-residential roster. During the interview, the respondent is asked about household members (co-residential household roster) and about non-resident members of the household (non-residential roster). The respondents identify their relationship with co-residential and non-residential household members, and we categorize them as ‘in a partnership’ if they name a wife/husband or a lover/partner as a co-residential or non-residential member of the household. Using this information and the sex of the respondent we are able to identify whether a partnership is different-sex or same-sex. Our dependent variable is a categorical variable linked to the current partnership state of an individual, which is ‘0’ if the respondent is not in a partnership, ‘1’ if they are in a different-sex partnership, and ‘2’ if in a same-sex partnership. Since these questions are included in the survey every year, the partnership state (i.e. in a partnership or not) and type of partnership (i.e. different-sex or same-sex) can vary over time. Although our theoretical framework is linked to understanding partnership formation, the data set lacks a variable on the timing of partnership formation, which limits our ability to model partnership formation directly as an event; instead, we focus on the partnership states that individuals are observed in over time.

Our sample includes individuals who were interviewed every year from 2003 to 2011, for whom information on partnership states over time is available, leaving us with a sample of 5,729 individuals (from an original 8,984 in 1997). In terms of confounding variables, we include controls for socio-demographic characteristics that can influence both the risk of partnership outcomes and the probability of having access to Internet. Hence, as well as *age* (and *age^2^*) to account for age effects for partnership transitions, we include *sex* (female = ‘1’), *race* (White, Black, Hispanic, and Other), *region* of residence (North East, North Central, South, and West), whether the respondent lives in a rural or *urban area* as per the US Census definition. We include two family background characteristics: *parents’ education* (less than high school, high school diploma, or more than high school), and *family income in 1997*. We also incorporate individual characteristics on the *level of education* (less than high school, high school diploma, some college, college degree or more) and whether *enrolled in school*, *income from job in past year* (log scale), and number of *weeks unemployed per year*. Finally, in order to consider factors that might influence the use of the Internet for online dating or the search for partners, we also consider *number of children in household*. The final sample size—excluding those without information for the control variables—is 5,513 respondents, of which 52.6 per cent are women and 47.4 per cent are men.

After presenting some descriptive statistics on the sample used in the analysis, we show multilevel multinomial logistic regression models we estimated to study the association between Internet access and being in either a different-sex or same-sex partnership. The models were estimated using Stata/MP 16.0 with the command *gllamm* (with a mlogit link). In our models recurrent partnership states are nested within individuals (Barber et al. 2000) and multilevel models allow us to introduce random effects, which represent individual-specific unobservables. We follow individuals in the sample over time, with a two-level hierarchical structure in which partnership states are clustered into individuals. Our general estimation approach is as follows:

(1) $$\log\left(\frac{P_{itj}}{P_{it0}}\right)=\alpha_{\;jt}+{\text{β}}_{j}Internet\_Access_{it}+\gamma_{j}x_{it}+\;e_{ij}$$

where *j* refers to the different states (not in a partnership, in a different-sex partnership, in a same-sex partnership) for each individual *i* in each time interval *t*, and $P_{it0}$ is the probability that a person is not in a partnership at time *t*. To examine our hypothesis about the age-dependent association between Internet access and partnership formation across the young adult life course, in our full model we include the interactions between Internet access and age and between Internet access and age^2^, as follows:

(2) $$\begin{array}{rl} \log\left(\frac{P_{itj}}{P_{it0}}\right)= & \alpha_{\;jt}+{\text{β}}_{1j}Internet_{Access\;\;it}+\;{\text{β}}_{2j}Age_{it}\\ & +\;{\text{β}}_{3j}Age_{it}^{2}\;\\ & +{\text{β}}_{4j}Internet_{Access\;\;it}\times Age_{it}\\ & +\;{\text{β}}_{5j}Internet_{Access\;\;it}\times Age_{it}^{2}\\ & +\;\gamma_{j}x_{it}+\;e_{ij} \end{array}$$

### Descriptive findings

Respondents in our sample were born between 1980 and 1984. Therefore from 2003 to 2011 they were aged 18–31, a period in the young adult life course when individuals enter significant relationships and possibly get married. As we can see in Table 1, the proportion of respondents in a relationship increased substantially as the cohort aged, from 28 per cent in 2003 to 71.6 per cent in 2011. This increase can be observed for both different-sex and same-sex partnerships, where the proportion in each type of relationship more than doubled over the eight-year period. The number of respondents in same-sex relationships was considerably lower (ranging between 36 and 84 people each year) than the number in different-sex relationships.

**Table 1:** Partnership state and Internet access, United States, 2003–11 (NLSY97 cohort)

	In a different-sex partnership		In a same-sex partnership		Total in a partnership		Access to Internet		Access to Internet at home	
Year	*n*	%	*n*	%	*n*	%	*n*	%	*n*	%
2003	1,508	27.4	36	0.7	1,544	28.0	4,450	80.7	3,388	61.5
2004	1,957	35.5	39	0.7	1,996	36.2	4,409	80.0	3,352	60.8
2005	2,342	42.5	48	0.9	2,390	43.4	4,507	81.8	3,475	63.0
2006	2,705	49.1	60	1.1	2,765	50.2	4,640	84.2	3,625	65.8
2007	3,037	55.1	68	1.2	3,105	56.3	4,745	86.1	3,806	69.0
2008	3,311	60.1	76	1.4	3,387	61.4	4,547	82.5	3,952	71.7
2009	3,519	63.8	74	1.3	3,593	65.2	4,593	83.3	–	–
2010	3,732	67.7	77	1.4	3,809	69.1	4,651	84.4	–	–
2011	3,866	70.1	84	1.5	3,950	71.6	4,804	87.1	–	–

*N = *5,513.

*Source:* NLSY97 data.

Over the same time span, we observe an increase in the percentage of young adults in the sample having access to Internet. Table 1 shows that 80.7 per cent of the sample had access to Internet in 2003, and by 2011 this figure had increased to 87.1 per cent. The same growth can be seen in the rising proportion of those having Internet access at home: from 61.5 per cent in 2003 to 71.7 per cent in 2008.

Table 2 shows descriptive statistics for the control variables used in the analysis, with the upper panel for time-constant variables and lower panel for time-varying variables. The time-varying covariates show increases with age in educational level, income from work, number of children in the household, and weeks unemployed per year for the NLSY97 cohort.

**Table 2:** Control variables used in analysis of NLSY97 cohort, United States, 2003–11

*Time-constant variables*									
% female	52.6								
Race (%)									
White	49.5								
Black	27.0								
Hispanic	20.0								
Other	3.6								
Parents’ education (%)									
Less than high school	16.5								
High school diploma	33.8								
More than high school	49.7								
Family income 1997, average USD	46,427								
*Time-varying variables*									
Year	2003	2004	2005	2006	2007	2008	2009	2010	2011
Mean age	20.9	21.9	22.9	23.9	24.8	25.8	26.7	27.8	28.7
Region (%)									
North East	16.2	16.0	15.6	15.5	15.3	15.3	15.2	15.5	15.4
North Central	23.4	23.2	23.0	22.7	22.3	22.1	21.9	21.8	21.8
South	38.5	38.9	39.3	39.2	39.7	39.7	40.1	40.1	39.9
West	21.9	22.0	22.1	22.6	22.6	22.8	22.7	22.7	22.9
% urban	78.8	80.6	81.3	80.9	80.9	81.3	80.9	79.0	78.9
Level of education (%)									
Less than high school	20.5	18.3	18.0	17.8	17.6	17.6	17.5	17.4	17.3
High school diploma	73.3	69.0	63.1	58.0	52.9	50.5	48.8	47.2	46.2
Some college	2.2	4.2	5.2	5.8	6.4	6.6	7.0	7.4	7.7
College degree or more	3.9	8.6	13.7	18.3	23.1	25.3	26.8	28.0	28.8
% enrolled in school	38.1	31.1	25.4	20.3	16.5	15.0	14.5	13.7	12.2
Income from job past year, average USD (if working)	10,344	13,120	15,833	18,919	22,786	26,230	29,087	30,727	33,026
Weeks unemployed per year, average (if > 0)	11.2	11.4	10.7	10.6	10.6	12.5	17.1	18.5	17.6
Number of children in the household, average	0.28	0.35	0.43	0.51	0.59	0.68	0.75	0.84	0.91

*N* = 5,513.

*Source:* Authors’ analysis of NLSY97 data.

**Figure 1:**
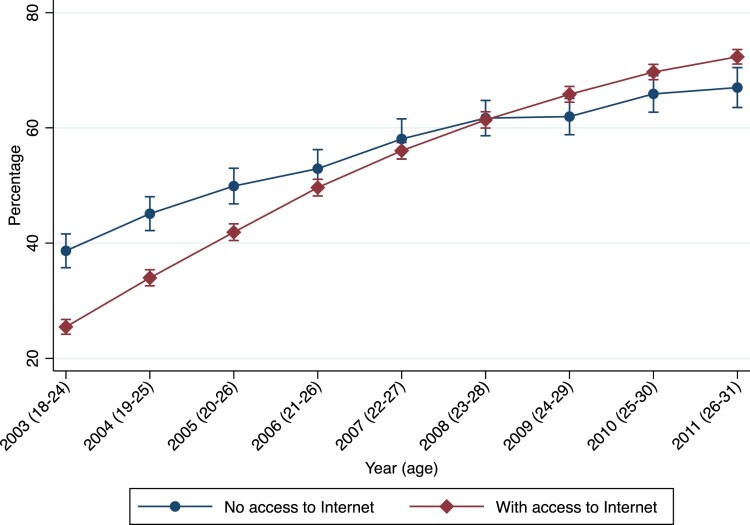
Predicted probability of being in a partnership by Internet access by year (age) of the NLSY97 cohort, United States *Note*: Vertical bars show 95 per cent confidence intervals. *Source*: NLSY97 data.

We start by examining the association between our two variables of interest—Internet access and partnership state—without any controls and without distinguishing between different-sex or same-sex partnerships (Figure 1). Until 2006 the predicted probability (estimated from a logistic regression) of being in a partnership was lower among those who *did* have access to the Internet (ages 21–26). In 2007 and 2008 the two groups almost overlapped, and from 2009 onwards (ages 24–29), Internet access was associated with a higher proportion of people being in a partnership. Hence, the relationship between Internet access and partnerships becomes positive as the cohort becomes older. Figure 1 suggests that Internet access might work in the same direction as higher education and socio-economic status, in terms of postponement of partnership formation followed by recuperation.

### Multilevel multinomial logistic regression analysis

Figure 1 does not consider any possible confounders, and the association between Internet access and partnership formation could be driven by other individual characteristics that are correlated with Internet use and independently predict individuals’ partnership states. Furthermore, individuals also experienced other transitions over this period (e.g. they became older and more educated), which may also have affected their partnership states. Table 3 presents results from multilevel multinomial logistic regression models that control for potential confounders, to analyse the association between Internet access and partnership states net of a range of controls. Our reference group is ‘not in a partnership’, and the two outcome groups are ‘in a different-sex relationship’ and ‘in a same-sex relationship’. Model (1) includes Internet access along with demographic (age, age^2^, sex, and race) and geographic (region of residence and urban vs rural location) controls. Model (2) further incorporates socio-economic characteristics: level of education, parents’ education, family income in 1997 (quintile), income from job in past year, number of weeks unemployed per year, and number of children in household. Finally, in model (3) we include the interactions between Internet access and age and between Internet access and age^2^, given that our hypothesis is an age-dependent association of Internet access and partnership formation. We report the exponentiated coefficients (odds ratios (ORs)) relative to the base outcome (not in a partnership) in Table 3.

**Table 3:** Multilevel multinomial logistic regression models of partnership, NLSY97 cohort, United States

*Dependent variable = In a partnership (ref: Not in a partnership)*	*Different-sex partnership Odds ratio (standard error)*			*Same-sex partnership Odds ratio (standard error)*		
	(1)	(2)	(3)	(1)	(2)	(3)
Internet access	0.632***	1.039	0.184***	0.698**	1.112	0.149***
	(0.058)	(0.091)	(0.065)	(0.102)	(0.168)	(0.106)
Age	7.855***	6.107***	4.859***	7.262***	6.348***	4.467***
	(0.421)	(0.345)	(0.493)	(0.660)	(0.595)	(0.859)
Age^2^	0.946***	0.953***	0.955***	0.947***	0.952***	0.963***
	(0.003)	(0.004)	(0.007)	(0.006)	(0.006)	(0.013)
Internet access × Age	–	–	1.352***	–	–	1.563**
			(0.150)			(0.335)
Internet access × Age^2^	–	–	0.997	–	–	0.985
			(0.008)			(0.015)
Female	3.678***	2.863***	3.253***	3.038***	3.321***	3.766***
	(0.581)	(0.420)	(0.389)	(0.543)	(0.570)	(0.561)
Race (ref: White)						
Black	0.032***	0.005***	0.006***	0.039***	0.007***	0.007***
	(0.007)	(0.001)	(0.001)	(0.009)	(0.002)	(0.001)
Hispanic	0.654**	0.317***	0.338***	0.692*	0.345***	0.369***
	(0.123)	(0.056)	(0.055)	(0.151)	(0.074)	(0.075)
Other	0.020***	0.025***	0.032***	0.013***	0.015***	0.019***
	(0.011)	(0.007)	(0.008)	(0.009)	(0.006)	(0.008)
Region (ref: North East)						
North Central	6.135***	2.213***	1.916***	2.473***	0.915	0.795
	(1.496)	(0.400)	(0.341)	(0.681)	(0.202)	(0.174)
South	4.494***	3.210***	2.831***	2.143***	1.564**	1.382*
	(0.795)	(0.466)	(0.406)	(0.444)	(0.283)	(0.248)
West	3.693***	2.566***	2.372***	1.656**	1.126	1.041
	(0.702)	(0.385)	(0.356)	(0.378)	(0.220)	(0.204)
Urban area	1.244**	1.269***	1.240**	2.127***	2.019***	1.978***
	(0.114)	(0.112)	(0.107)	(0.330)	(0.311)	(0.302)
Enrolled in school	–	0.319***	0.313***	–	0.402***	0.391***
		(0.032)	(0.027)		(0.061)	(0.056)
Level of education (ref: < High school)						
High school diploma	–	0.335***	0.383***	–	0.281***	0.320***
		(0.054)	(0.063)		(0.055)	(0.064)
Some college	–	0.474***	0.507***	–	0.194***	0.207***
		(0.102)	(0.115)		(0.063)	(0.069)
College degree or more	–	0.296***	0.275***	–	0.131***	0.122***
		(0.076)	(0.058)		(0.040)	(0.032)
Parents’ education (ref: < High school)						
High school diploma	–	0.899	0.943	–	0.466***	0.490***
		(0.150)	(0.163)		(0.098)	(0.106)
More than high school	–	0.279***	0.291***	–	0.258***	0.269***
		(0.053)	(0.053)		(0.059)	(0.059)
Family income in 1997 (ref: 1st quintile)						
2nd quintile	–	1.606**	1.488**	–	3.581***	3.334***
		(0.343)	(0.283)		(0.953)	(0.823)
3rd quintile	–	1.369	1.482*	–	1.417	1.542
		(0.304)	(0.304)		(0.415)	(0.432)
4th quintile	–	0.932	1.044	–	0.969	1.092
		(0.244)	(0.226)		(0.317)	(0.318)
5th quintile	–	0.113***	0.121***	–	0.211***	0.227***
		(0.029)	(0.026)		(0.067)	(0.063)
Income missing	–	0.299***	0.240***	–	0.560**	0.451***
		(0.075)	(0.051)		(0.164)	(0.118)
Log of income from job past year	–	1.080***	1.081***	–	1.047***	1.049***
		(0.011)	(0.011)		(0.016)	(0.017)
Weeks unemployed per year	–	0.997	0.997	–	1.008	1.008
		(0.003)	(0.003)		(0.005)	(0.005)
Number of children in household	–	6.988***	7.284***	–	3.001***	3.130***
		(0.498)	(0.509)		(0.293)	(0.302)
Constant	0.000***	0.000***	0.001***	0.000***	0.000***	0.000***
	(0.000)	(0.000)	(0.000)	(0.000)	(0.000)	(0.000)
Random intercept variance	27.9	28.1	28.1	27.9	28.1	28.1
	(0.716)	(0.755)	(0.756)	(0.716)	(0.755)	(0.756)
*N*	49,617 (5,513 individuals over nine years)					

* *p*<0.10, ** *p*<0.05, *** *p*<0.01.

*Source:* As for Table 2.

Model (1) in Table 3 shows that Internet access is associated with lower odds of being in a partnered state, for both different-sex and same-sex partnerships. The average marginal effect (AME) of Internet access from model (1) is −0.026, that is, Internet access is associated with a 2.6 percentage point lower probability of being in a different-sex partnership. For same-sex partnerships, given the smaller baseline probability of being in these unions in the sample (1.13 per cent), the AME is −0.0005. As expected, the odds of being in a partnership increase with age and are higher for women than for men. Model (2), which includes socio-economic and demographic characteristics, shows that Internet access is associated with slightly higher odds of being in a partnership both for those in a different-sex partnership and those in a same-sex partnership, although these associations in model (2) are not significant.

Finally, in model (3), which includes the interaction term between age and access to Internet (and age^2^ and Internet access), we see that the main effect of Internet access is less than 1.0 (lower odds) and that of age is greater than 1.0 (higher odds). The AME of Internet access from model (3) is −0.077 for different-sex partnerships and −0.005 for same-sex partnerships. Notably, the interaction term between age and Internet access is greater than 1.0 and significant for both different-sex and same-sex partnerships. This suggests, consistent with the preliminary descriptive results in Figure 1, that the marginal effect of Internet access for being in a partnership state increases with age. Expressed in terms of predicted probabilities, while at age 20 the probability of entering a different-sex partnership is nine percentage points lower for those with Internet access than those without, by age 26, a crossover emerges, such that Internet access positively predicts partnership. For example, by age 28 the predicted probability of being in a different-sex partnership is 3.3 percentage points higher for those with Internet access. Similarly, at age 20 the probability of being in a same-sex partnership is 0.2 percentage points lower for those with Internet access, but by age 28 this is 0.04 percentage points higher. These patterns are also consistent with those in a logistic model with a dichotomous outcome (‘in a partnership’) (see Table A12 in the supplementary material).

We further replicated the models stratifying by sex, even though in that case we could not distinguish between same-sex and different-sex partnerships, given the much smaller sample size. The results—reported in the supplementary material (Table A1)—showed similar findings to the aggregate analysis, with a positive interaction indicating increased odds with age for Internet access. We also replicated the analysis separately for different types of co-residential unions (i.e. cohabitations and marriages). In the NLSY97, given the younger age of the sample (compared with the CPS), the proportion of people cohabiting is similar to the proportion married. The regression results (not shown) confirmed that the findings are similar among those who marry and those who cohabit, except for non-significant results for same-sex cohabiting relationships.

## Empirical cross-sectional analysis using Current Population Survey data

### Data and methods

Our second set of analyses was performed using the CPS data. The CPS is a cross-sectional household survey in the US that collects monthly data on several different topics, including demographic and socio-economic information. In 1997 the CPS started collecting data on digital connectivity in a Computer and Internet Use supplement (CIS), and this information is available for 12 years, covering 1997–98, 2000–01, 2003, 2007, 2009–13, and 2015. In this supplement, all the respondents in the household were asked whether they had access to Internet at home and if the household had an Internet connection. For the purpose of our analysis, we use this information to build our independent variable, that is, having access to the Internet at home. This variable is equal to ‘1’ if the household has an Internet connection or (when the answer to that question is missing) if another member of the household reports they have access to the Internet from home.

The partnership status of the main respondent is established using the household roster, from the presence of a spouse or unmarried partner in the same household. Like for the NLSY97 data, we can distinguish between different-sex and same-sex unions based on the sex of the household head and the partner (if present). Therefore, our dependent variable using the CPS data is the same as for the NLSY97 analysis (‘0’ if the respondent is not in a partnership, ‘1’ if they are in a different-sex relationship, and ‘2’ if in a same-sex relationship). Unlike the NLSY97, however, the CPS is a cross-sectional data set and we are unable to observe transitions in partnership states over time. However, the CPS provides us with repeated cross-sections of multiple cohorts over a longer duration (1997–2015) over the course of Internet diffusion to compare with the NLSY97 findings.

The sample includes individuals that were interviewed in the CIS and had answered the questions on Internet access. We start with a sample of 952,892 individuals aged 15 years and older, over the 12 survey years, and we are left with 619,158 after excluding those for whom no data on Internet access is available due to their exclusion from the CIS (descriptive statistics for the sample with and without Internet access information are presented in Table A11, supplementary material). We include several control variables, similar to those in the NLSY97 analyses: *age* at interview (and *age^2^*), *sex*, *race* (White, Black, Hispanic, Asian, American Indian, and Other/Mixed), US state fixed effects, whether living in a *metro area*, the *level of education* (less than high school, high school diploma, some college, college degree or more), *family income in 1997* (< $25,000, $25,000–49,999, $50,000–74,999, $75,000 and over), *weekly earnings at current job*, number of *continuous weeks unemployed*, and *number of children in household*. Descriptive statistics on the partnership status of the respondent, access to Internet, and control variables over the years in our selected sample are reported in the supplementary material (Tables A2 and A3). We use multinomial logistic regression models to investigate the relationship between Internet access and partnership status, distinguishing between different-sex and same-sex unions. These models were estimated using Stata 16.0 with the *mlogit* command.

### Descriptive findings

**Figure 2:**
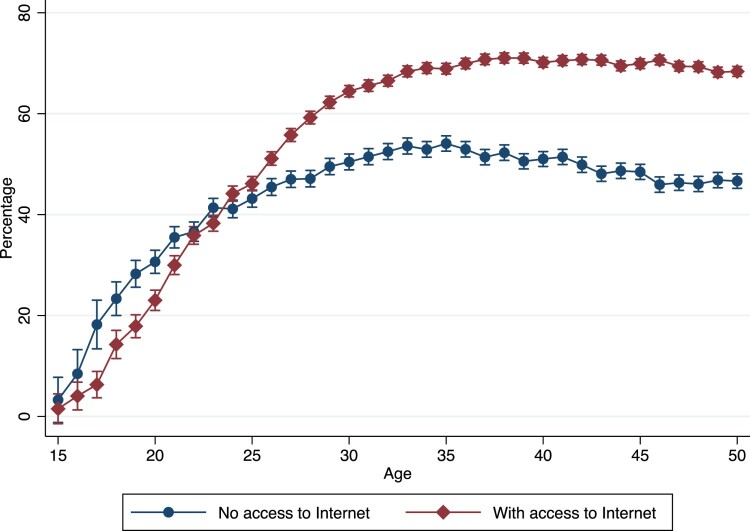
Predicted probability of being in a partnership by Internet access and age (CPS), United States, pooled data from 1997 to 2015 *Note*: Vertical bars show 95 per cent confidence intervals. *Source*: CPS data.

Figure 2 shows the predicted probability of being in a partnership by age and Internet access, estimated from a logistic regression model with age dummies and Internet access without further controls. The patterns are similar to the NLSY97 (Figure 1), except that unlike the NLSY97, which is limited to young adults, the CPS provides a sample that covers older ages as well (from 15 up to 90 years old). In Figure 2, we show results only up to age 50 for ease of visualization and due to smaller sample sizes at older ages, especially in the ‘no Internet access’ category (the regression models, however, are based on the whole age range included in the CPS data). We observe how at younger ages the predicted probability of being in a co-residential union is lower for those with Internet access at home compared with those without (until ages 22–24), but becomes higher after age 25 and remains higher. We replicated the same analysis across different years (see Figure A1, supplementary material), and the results showed how the association between Internet access and being in a partnership is consistent over the different periods for which the CPS data are available.

### Multinomial logistic regression analysis

We ran multinomial logistic regression models controlling for confounders that could influence the association between Internet access and partnership status, and to distinguish between different-sex and same-sex unions. Table 4 reports the exponentiated coefficients (ORs) from three different models: Model (1) includes Internet access, age, and age^2^ (all ages included), sex (female = ‘1’), race, whether living in a metro area, and US state and year fixed effects; model (2) adds level of education, family income, weekly earnings from current job, number of weeks of continuous unemployment, and number of children in the household; and model (3) includes the interaction terms between age and Internet access and between age^2^ and Internet access.

**Table 4:** Multinomial logistic regression models of partnership, CPS data, United States, 1997–2015

*Dependent variable = In a partnership* *(ref: Not in a partnership)*	*Different-sex partnership* *Odds ratio (standard error)*			*Same-sex partnership Odds ratio (standard error)*		
	(1)	(2)	(3)	(1)	(2)	(3)
Internet access	2.488***	1.699***	1.390***	3.661***	2.477***	0.041***
	(0.016)	(0.013)	(0.073)	(0.262)	(0.187)	(0.022)
Age	1.106***	1.053***	1.048***	1.172***	1.178***	1.038*
	(0.001)	(0.001)	(0.002)	(0.012)	(0.013)	(0.022)
Age^2^	0.999***	1.000***	1.000***	0.998***	0.998***	0.999***
	(0.000)	(0.000)	(0.000)	(0.000)	(0.000)	(0.000)
Internet access × Age	–	–	1.001	–	–	1.169***
			(0.002)			(0.029)
Internet access × Age^2^	–	–	1.000***	–	–	0.999***
			(0.000)			(0.000)
Female	0.375***	0.344***	0.345***	0.783***	0.843***	0.843***
	(0.002)	(0.002)	(0.002)	(0.037)	(0.040)	(0.040)
Race (ref: White)						
Black	0.512***	0.510***	0.508***	0.236***	0.327***	0.325***
	(0.005)	(0.006)	(0.006)	(0.027)	(0.037)	(0.037)
Hispanic	1.337***	1.229***	1.216***	0.627***	0.946	0.917
	(0.014)	(0.015)	(0.015)	(0.058)	(0.090)	(0.087)
Asian	1.293***	1.293***	1.305***	0.385***	0.405***	0.414***
	(0.024)	(0.026)	(0.026)	(0.065)	(0.069)	(0.070)
American Indian	0.729***	0.696***	0.691***	0.611	0.79	0.791
	(0.021)	(0.022)	(0.022)	(0.188)	(0.243)	(0.243)
Other/Mixed	0.929***	0.973	0.976	0.692**	0.82	0.827
	(0.020)	(0.023)	(0.023)	(0.125)	(0.148)	(0.149)
Metro area (ref: Not in metro area)						
Central city	0.566***	0.545***	0.546***	1.866***	1.418***	1.432***
	(0.005)	(0.006)	(0.006)	(0.164)	(0.127)	(0.128)
Outside central city	0.952***	0.777***	0.777***	1.274***	1.013	1.014
	(0.008)	(0.007)	(0.007)	(0.112)	(0.090)	(0.090)
Missing/Unknown	0.868***	0.813***	0.813***	1.243**	1.107	1.11
	(0.008)	(0.008)	(0.008)	(0.109)	(0.098)	(0.098)
Level of education (ref: < High school)						
High school diploma	–	0.981*	0.971***	–	0.865	0.851
		(0.010)	(0.010)		(0.110)	(0.108)
Some college	–	0.772***	0.765***	–	1.032	1.018
		(0.008)	(0.008)		(0.128)	(0.126)
College degree or more	–	0.649***	0.643***	–	1.246*	1.238*
		(0.008)	(0.008)		(0.155)	(0.154)
Family income in 1997 (ref: < $25,000)						
$25,000–49,999	–	2.361***	2.355***	–	1.474***	1.471***
		(0.020)	(0.020)		(0.122)	(0.122)
$50,000–74,999	–	4.385***	4.385***	–	2.278***	2.268***
		(0.045)	(0.045)		(0.199)	(0.199)
$75,000 and over	–	8.764***	8.789***	–	5.249***	5.157***
		(0.098)	(0.098)		(0.428)	(0.422)
Missing	–	2.362***	2.369***	–	0.998	0.997
		(0.027)	(0.027)		(0.144)	(0.143)
Weekly earnings, current job	–	1.000***	1.000***	–	1.000***	1.000***
		(0.000)	(0.000)		(0.000)	(0.000)
Continuous weeks unemployed	–	1.000	1.000	–	1.006***	1.006***
		(0.000)	(0.000)		(0.002)	(0.002)
Number of children in household	–	1.916***	1.914***	–	0.676***	0.665***
		(0.007)	(0.007)		(0.026)	(0.025)
Constant	0.252***	0.274***	0.335***	0.000***	0.000***	0.002***
	(0.009)	(0.011)	(0.016)	(0.000)	(0.000)	(0.001)
*N*	619,158					

* *p*<0.10, ** *p*<0.05, *** *p*<0.01.

*Note:* All the specifications include US state and year fixed effects.

*Source:* Authors’ analysis of CPS data.

Model (1) in Table 4 shows that the odds of being in a different-sex or same-sex union are higher for those with Internet access. The AME of Internet access for different-sex partnerships from model (1) is 0.188, that is, Internet access is associated with an 18.8 percentage point higher probability of being in a different-sex partnership; it is also associated with a 0.2 percentage point higher probability of being in a same-sex union (AME = 0.002). On the inclusion of additional controls for socio-economic status (model (2)), Internet access remains associated with higher odds for both partnership outcomes. On comparing models (1) and (2), the AME remains positive and statistically significant, although it diminishes in magnitude to 0.09 for different-sex partnerships and 0.0018 for same-sex partnerships. Finally, in model (3), when we include the interaction between age and Internet access (and between age^2^ and Internet access), the odds ratio of the Internet access term for different-sex unions (OR = 1.390, *p *< 0.01, AME = 0.089) remains greater than 1.0, whereas it is smaller than 1.0 for same-sex unions (OR = 0.041, *p *< 0.01, AME = −0.05). The interaction term with age is greater than 1.0 for both different-sex and same-sex partnerships but statistically significant only for same-sex partnerships. The results are consistent with those in a logistic model (see Table A13, supplementary material) with a dichotomous outcome (‘in a partnership’). A visualization of the interaction from the dichotomous outcome (being in a partnership) estimated using a logistic regression with controls is reported in Figure A2 (supplementary material). Like the NLSY97 results, the CPS patterns showed that the association between Internet access and being in a partnership strengthens with age, net of controls, and emerges as a positive association after the mid-20s. The age dependency of the association between Internet access and partnership outcomes across both data sets also explains the difference in the main effect of Internet access in the NLSY97 (a younger sample on average) compared with the CPS (an older sample).

We further conducted the analyses separately by sex. This showed, first, that the predicted probability of being in a union (Figure A3, supplementary material) is higher among men than among women at all ages, independently of Internet access. Second, the crossover age at which Internet access is associated with a higher probability of being in a union is slightly higher for men than for women. This result seems to be in line with the fact that women enter unions earlier than men and possibly that they are ready to settle down earlier, in which case the positive relationship with Internet access shows up earlier in the life course. The positive association at older ages fades among women when they approach age 50, but it stays positive among men. When we replicated the regression analyses stratifying by sex and same-sex vs different-sex relationships (Tables A4 and A5, supplementary material) the results showed similar findings to the aggregate analysis.

We also analysed the results separately by different types of unions (i.e. cohabitation and marriage) from the CPS (results not shown). In the CPS sample, the proportion of respondents who are cohabiting (4.8 per cent of the whole sample) is much lower compared with those who are married (50.8 per cent of the whole sample), especially for earlier birth cohorts. As expected, the picture for marriage closely resembles the general results, given that 91.4 per cent of the co-residential partnerships recorded in our sample are marriages. The predicted probability of being in a cohabitation increases until age 23–24 and then consistently declines at older ages, predominantly because people move into marriages. However, we still observe a positive interaction in the same direction between age and Internet access (although this is not statistically significant): the predicted probability of being in a cohabitation is higher for those without Internet access at younger ages (up to age 20) and then it becomes higher for those with access to the Internet (up to age 30).

Finally, all the analyses shown so far have been based on Internet access from home. We repeated the analysis using all the available information on Internet access at any location (e.g. school, or library). The results (reported in Figure A4, supplementary material) showed that independently of how the respondents in our sample access the Internet, the association with being in a partnership and the interaction with age do not change. We note that the predicted probability of being in a union is higher for those with Internet access at ages after 27, later than when using the Internet access at home variable, and that the difference between the two groups at older ages is less marked than in the main analysis considering only Internet access at home, which would be in line with the expectation that Internet access at home affords easier access and thus differences are more marked.

## Robustness checks

For both data sets we performed additional analyses as robustness checks (results in supplementary material). First, in order to measure Internet access *prior to* partnership formation more clearly, we replicated the main analysis in the NLSY97 using a lagged version of Internet access. This check also addressed potential concerns linked to reverse causality, in case Internet access is enabled through partnership formation by pooling resources with a partner. In this way we measured Internet access prior to partnership formation and evaluated the association between access to the Internet at *t*−*1* and partnership formation at time *t*. The results (Table A6, supplementary material) showed very similar findings to the main analysis: the interaction term between Internet access and age was still positive and significant. As additional robustness checks we also ran lagged models measuring Internet access at *t*−2 and at *t = *2003 (beginning of the observation window), as well as fixed effects models (only on individuals whose Internet access changes over time; see Table A7, supplementary material). These additional analyses further confirmed the results in the main analysis.

Second, to examine if our Internet access effect is capturing a general technology effect linked to household economic status, we compared Internet access with TV viewing. The NLSY97 data include a question on weekly hours of TV watched for the years 2002 and 2007–11. Hence, we replicated the analysis—without distinguishing between different-sex and same-sex partnerships—for this subset of years including *weekly hours of TV watched* as an additional control variable, but we also ran additional models substituting this variable for Internet access. The results (reported in Table A8, supplementary material) showed that when TV viewing is included as an additional control variable, it does not change the general patterns of the main NLSY97 results reported earlier (models (1) and (2)). However, watching TV itself is not significantly associated with partnership states, and there is no age interaction in this effect either, as there is with Internet access (model (3)).

Following a similar strategy to Fairlie et al. (2010), who examined the association between computer use and high school graduation with the CPS, we created a panel data set exploiting the rotation pattern of the CPS. Households in the CPS are interviewed every month for four months. Eight months later they are reinterviewed in each month of a second four-month period. Hence, we can link information on individuals in the CIS who are in their first rotation period to information from the same month in their second four-month rotation period a year later. Thus, a two-year panel can be created for a subset of the respondents in the CIS. Based on survey years available and information on Internet access, we obtained a subsample of 120,607 individuals and like the NLSY97 lagged approach where we measured Internet access prior to partnership, we used Internet access at time *t*−*1* to predict the probability of being in a partnership at time *t*. The results (in Table A9, supplementary material) confirmed the same pattern as the main analysis, with a significant, positive interaction of Internet access and age.

The final robustness check using the CPS also follows Fairlie et al.’s (2010) strategy. We estimated bivariate probit models in which equations for the probability of being in a partnership and the probability of having access to the Internet are estimated simultaneously. In the regression model to estimate Internet access, we also included a variable that indicates whether the respondent *directly uses the computer at work*. We would expect this variable to be associated with the probability of having access to the Internet but not directly with the probability of being in a partnership other than through the economic controls already included in the analysis (education, employment, and income). This variable is available only for the years 1997, 2001, and 2003, so we could run these models on only a subset of individuals (*n = *104,151). The results (reported in Table A10, supplementary material) confirmed those found in the main analyses using both the NLSY97 and the CPS.

## Discussion

This paper contributes to a burgeoning literature that explores the implications of the Internet for family and life-course outcomes (e.g. Rosenfeld and Thomas 2012; Bellou 2015; Dettling 2017; Rosenfield 2017; Billari et al. 2019). We analysed the relationship between Internet access and partnership formation, including both cohabitation and marriage, using individual-level longitudinal data from the NLSY97. This data set allowed us to follow a young adult cohort and examine how Internet access is associated with partnership transitions as the cohort grows older. We further analysed the association between Internet access and the probability of being partnered using repeated cross-sections, as well as a shorter two-year panel, with CPS data that cover a longer period of technology diffusion (1997 onwards) than the NLSY97. Although the potential role of the Internet for partnership formation is theoretically plausible and has attracted significant interest (both scholarly and public), nationally representative social and demographic surveys with information on digital connectivity and partnership history are surprisingly limited. Our work explored this interesting and important question by drawing on two data sources that contain both digital and demographic information, and by examining this association with similar controls for socio-economic and demographic confounders across different data sources, we assessed if the associations were consistent across them. We built on previous work by analysing the relationship between technology and both different-sex and same-sex partnerships (and both cohabitations and marriages) and by integrating a life-course perspective that explored whether the associations between technology and partnership were age-dependent.

Our findings across both data sources showed a consistent age-dependent association between Internet access and partnership states (in the NLSY97) or status (in the CPS) for both different-sex and same-sex partnerships, net of a wide range of socio-demographic confounders. While at younger ages we found a negative association, after the mid-20s, the relationship between Internet access and partnership status becomes positive. Following our theoretical framework, an interpretation of this negative association is that at younger ages Internet access provides individuals with the opportunity to expand their networks and meet new people, which may potentially postpone partnership formation. An alternative explanation is that at younger ages Internet access is used for purposes other than partner search, with Internet use linked to romantic or sexual behaviours varying by age. By the mid-20s, however, Internet access becomes positively associated with the propensity to partner, which is consistent with a life-course perspective in which technology can be a supportive and enabling force at ages when individual desires (or social norms) to settle down become stronger. Seen in this way, the Internet is not a driving force of partnership outcomes but a supportive agent, as has been suggested for the Internet and other demographic processes, such as migration decisions (Pesando et al. 2021). Existing work suggests a more significant role of Internet technologies for partnership formation among those in thin markets, for example LGB individuals. Our analyses in both the NLSY97 and CPS pointed in this direction, with the positive association of Internet access with age rising more steeply for same-sex partnerships, although the small sample sizes for same-sex partnerships (especially in the NLSY97) made these patterns difficult to estimate with precision. The fact that our findings were similar across the NLSY97 cohort and the CPS suggests that the age-dependent association between Internet access and partnership outcomes is not restricted to a specific cohort.

We acknowledge that our study suffers from a number of limitations. The data did not allow us to measure exactly how individuals use the Internet, and as a result, we could not examine the different likely mechanisms through which Internet access is associated with partnership outcomes (e.g. access to a wider pool of partners, improved information availability, or more frequent or intimate communication). Also, neither data source collects information on dating histories that would have allowed us to examine the mechanisms linked to expanded partner pools (e.g. increased dating frequency). A further limitation is that of potential reverse causality whereby partnership formation could enable access to the Internet for some individuals, through pooling partners’ incomes, rather than the other way around. We attempted to address this partially with the longitudinal data structure of the NLSY97, where time-varying information on Internet access and partnership state is available and we could also measure Internet access prior to partnership formation. In further robustness checks, by creating a one-year rotating CPS panel, we were also able to measure Internet access in the preceding year, but we recognize that this still provided us with only a short observation window. Unlike the CPS, which covers a longer period of time, a limitation of the NLSY97 data is that we were unable to capture the diffusion of the Internet over a longer period, as the Internet access question was only asked from 2003 onwards, when a significant fraction of the users already has access. While we controlled for several socio-demographic confounders in our analyses, it is plausible that Internet non-users are a select group on other unmeasured socio-economic or other characteristics (e.g. personality traits) with implications for partnership formation, which precludes us from making causal claims about Internet access. Assuming that unmeasured socio-economic confounding remains, an alternative interpretation of our finding is that the persistent gap between Internet users and non-users that we observed reflects how Internet access may serve as an additional factor that underpins the socio-economic gradient in partnership formation.

Despite these limitations, we believe our work makes a number of contributions that extend previous literature. First, we make a theoretical contribution by describing different mechanisms—including improved access to information and better communication and connectivity—that extend beyond considering only Internet dating for understanding the implications of Internet technologies for partnering processes. Second, we develop hypotheses that integrate perspectives from search, choice overload, and life-course theories to evaluate the direction of the associations between family and technology variables. Work by Bellou (2015) and Rosenfeld (2017) has shown a positive association between the Internet and partnership formation. While Bellou (2015) found higher marriage rates in US counties with better broadband availability, Rosenfeld (2017) found that the transition to partnership was faster for heterosexual couples who met online compared with those who met offline. Using different data from ours, Rosenfeld and Thomas (2012) also reported the probability of being partnered to be higher for those with Internet access net of controls. Our findings are consistent with these studies, but further highlight the importance of considering a life-course perspective that recognizes that the role of technology may vary by age. As digital technologies continue to permeate different domains of our lives, our work speaks to the need to better understand the impacts of these technologies on key life-course decisions across different contexts. Such research would benefit from a deeper integration of measures of digital technology use in socio-demographic surveys, as well as richer qualitative sources that explore the mechanisms linking how individuals use technologies in ways that affect demographic outcomes.

## Supplementary Material

Supplementary Material
