# Supplementary material for: Internet access and partnership formation in the United States
Source: Popul Stud (Camb). 2021 Nov 23;76(3):427–45. doi: 10.1080/00324728.2021.1999485 (PMC9621102; doi:10.1080/00324728.2021.1999485)

Online Appendix to Internet access and partnership formation in the United States. Maria Sironi and Ridhi Kashyap. *Population Studies.* 2021.

Table A1. Multinomial multilevel regression models (NLSY97) - By Gender

Y = being in a partnership (Ref: No)	Men			Women		
	(1)	(2)	(3)	(1)	(2)	(3)
Internet Access	1.203***	0.794***	0.128***	1.084	0.672***	0.053***
	(0.056)	(0.050)	(0.040)	(0.056)	(0.044)	(0.012)
Age	131.794***	271.178***	157.245***	126.477***	283.448***	1027.444***
	(15.557)	(25.775)	(21.348)	(11.048)	(24.991)	(138.772)
Age^2	0.947***	0.871***	0.882***	0.916***	0.855***	0.774***
	(0.002)	(0.003)	(0.008)	(0.002)	(0.003)	(0.005)
Internet Access*Age			1.534***			1.031
			(0.179)			(0.078)
Internet Access*Age^2			0.993			1.048***
			(0.009)			(0.006)
N	23,517 (2,613 men over 9 years)			26,100 (2,900 women over 9 years)		

* $p<0.10$, ** $p<0.05$, *** $p<0.01$. Model (1) controls for gender, ethnicity, region, urban; Model (2): (1) + education, parents' education, family income, income from work, weeks of unemployment, number of children in the household; Model (3): (2) + Internet access * age + Internet access * age^2.

Table A2. Partnership Status and Internet Access (CPS)

	In a different-sex partnership		In a same-sex partnership		Total in a partnership		Access to Internet at Home	
Year	N	Weighted %	N	Weighted %	N	Weighted %	N	Weighted %
1997	27,251	56.4	45	0.09	27,296	56.5	8,620	18.1
1998	27,229	56.2	50	0.10	27,279	56.3	12,709	26.4
2000	27,093	56.5	53	0.10	27,146	56.6	19,924	41.6
2001	31,908	55.8	96	0.18	32,004	55.9	28,864	50.5
2003	31,574	55.9	109	0.19	31,683	56.1	30,911	54.8
2007	29,786	54.4	148	0.26	29,934	54.6	33,930	61.9
2009	29,789	54.0	166	0.30	29,955	54.3	37,539	68.7
2010	29,635	54.0	206	0.34	29,841	54.3	38,804	71.1
2011	29,264	54.3	245	0.44	29,509	54.7	37,898	70.5
2012	29,156	53.8	239	0.41	29,395	54.2	40,462	74.8
2013	21,632	53.6	194	0.45	21,826	54.0	29,933	74.2
2015	28,106	53.2	313	0.63	28,419	53.8	38,462	73.4
Total	342,423	54.8	1,864	0.30	344,287	55.1	358,056	57.9

N=619,158. Source: CPS Data.

Table A3. Control Variables, Weighted (CPS)

Year		1997	1998	2000	2001	2003	2007	2009	2010	2011	2012	2013	2015	Total
% Female		40.0	41.8	44.5	46.2	46.7	48.7	48.6	49.4	49.2	49.6	49.6	49.3	47.1
Mean Age		48.2	48.4	48.7	48.7	48.4	49.0	49.6	49.8	50.0	50.3	50.5	50.8	49.4
Race (%)														
	White	75.3	75.1	74.4	73.8	72.1	70.3	69.7	69.6	69.5	68.2	68.0	66.9	71.0
	Black	12.1	12.1	11.9	12.0	11.8	12.3	12.5	12.4	12.4	12.4	12.4	12.7	12.3
	Hispanic	9.0	9.0	9.3	9.7	10.8	11.9	12.0	12.2	12.3	13.1	13.0	13.6	11.4
	Asian	2.8	-	-	-	3.5	3.8	3.9	3.9	4.0	4.3	4.5	4.6	3.0
	American Indian	0.7	0.8	0.8	0.8	0.5	0.5	0.7	0.6	0.6	0.7	0.7	0.6	0.7
	Other/Mixed	-	3.1	3.6	3.7	1.2	1.3	1.2	1.3	1.3	1.4	1.5	1.6	1.7
Region (%)														
	North East	19.4	19.0	19.3	19.4	19.1	18.3	18.3	18.2	18.0	17.8	17.8	17.6	18.5
	North Central	23.4	23.6	23.1	23.5	23.1	22.7	22.3	22.3	22.3	22.3	22.0	21.8	22.7
	South	35.7	36.0	36.2	35.9	36.0	36.7	37.1	37.2	37.5	37.7	37.8	38.0	36.8
	West	21.5	21.5	21.4	21.3	21.8	22.4	22.4	22.3	22.2	22.3	22.5	22.6	22.0
% Not in metro area		19.5	19.4	19.4	19.1	18.8	15.9	15.8	16.0	15.9	15.5	15.6	14.1	17.0
Level of Education (%)														
	Less than High School	17.6	17.4	16.6	16.0	15.0	12.8	12.5	12.0	11.8	11.4	11.1	10.4	13.6
	High School	31.7	31.3	30.7	30.7	30.4	29.9	29.3	29.2	29.3	28.5	28.4	27.4	29.7
	Some College	26.0	25.9	26.4	26.9	26.8	28.1	28.2	28.5	28.5	28.8	28.6	29.2	27.7
	College Degree or more	24.7	25.4	26.4	26.4	27.8	29.2	30.0	30.3	30.4	31.4	31.9	33.0	29.0
Family Income (%)														
	< $25,000	34.9	31.2	27.1	26.1	24.4	20.9	22.2	28.9	28.4	27.9	27.2	24.7	26.9
	$25,000-49,999	27.4	27.0	25.3	24.5	24.0	21.6	21.8	27.5	27.1	26.7	26.0	25.8	25.4
	$50,000-74,999	14.2	14.6	14.7	14.9	14.6	14.6	14.7	17.8	18.1	17.9	18.0	18.3	16.1
	$75,000 and over	11.7	13.5	16.4	17.5	17.6	20.4	21.0	25.9	26.4	27.5	28.8	31.3	21.7
	Missing	11.9	13.6	16.5	17.0	19.4	22.5	20.3	-	-	-	-	-	10.0
Avg. Earnings per Week (if working)		619.4	658.7	707.0	719.6	760.3	846.3	867.0	870.5	888.8	908.7	924.8	961.6	814.7
Avg. # Weeks Consecutive Unemployment (if >0)		18.6	15.6	15.0	14.6	21.3	18.5	29.2	37.7	39.3	39.1	34.1	25.6	28.8
Number of Children in the HH		0.82	0.81	0.79	0.78	0.77	0.74	0.74	0.74	0.76	0.73	0.73	0.72	0.76

N=619,158. Source: CPS Data.

Table A4. Multinomial multilevel regression models - MEN (CPS)

Y = being in a partnership (Ref: No)	Different-sex Partnership			Same-sex Partnership		
	(1)	(2)	(3)	(1)	(2)	(3)
Internet Access	2.824***	1.930***	1.487***	4.355***	2.807***	0.010***
	(0.026)	(0.021)	(0.115)	(0.483)	(0.327)	(0.009)
Age	1.109***	1.037***	1.030***	1.228***	1.249***	1.036
	(0.002)	(0.002)	(0.002)	(0.021)	(0.024)	(0.036)
Age^2	0.999***	1.000***	1.000*	0.998***	0.998***	0.999*
	(0.000)	(0.000)	(0.000)	(0.000)	(0.000)	(0.000)
Internet Access*Age			0.999			1.254***
			(0.003)			(0.052)
Internet Access*Age^2			1.000***			0.998***
			(0.000)			(0.000)
N	325,442					

* p<0.10, ** p<0.05, *** p<0.01. All the specifications include dummies for US State and Year. Model (1) controls for gender, ethnicity, US state, metro area; Model (2): (1) + education, family income + weekly earnings + weeks of continuous unemployment + number children in the household; Model (3): (2) + Internet access * age + Internet access * age^2

Table A5. Multinomial multilevel regression models - WOMEN (CPS)

Y = being in a partnership (Ref: No)	Different-sex Partnership			Same-sex Partnership		
	(1)	(2)	(3)	(1)	(2)	(3)
Internet Access	2.122***	1.469***	1.482***	3.208***	2.236***	0.139***
	(0.020)	(0.016)	(0.111)	(0.301)	(0.222)	(0.095)
Age	1.108***	1.057***	1.057***	1.132***	1.126***	1.04
	(0.002)	(0.002)	(0.002)	(0.015)	(0.016)	(0.028)
Age^2	0.999***	0.999***	0.999***	0.998***	0.998***	0.999***
	(0.000)	(0.000)	(0.000)	(0.000)	(0.000)	(0.000)
Internet Access*Age			0.993**			1.101***
			(0.003)			(0.035)
Internet Access*Age^2			1.000***			0.999**
			(0.000)			(0.000)
N	293,716					

* p<0.10, ** p<0.05, *** p<0.01. All the specifications include dummies for US State and Year. Model (1) controls for gender, ethnicity, US state, metro area; Model (2): (1) + education, family income + weekly earnings + weeks of continuous unemployment + number children in the household; Model (3): (2) + Internet access * age + Internet access * age^2

Table A6. Multinomial multilevel regression models (NLSY97) - Internet Access at t-1

Y = being in a partnership (Ref: No)	Different-sex Partnership			Same-sex Partnership		
	(1)	(2)	(3)	(1)	(2)	(3)
Internet Access [t-1]	0.565***	0.804**	0.168***	0.589***	0.796	0.097**
	(0.054)	(0.079)	(0.085)	(0.088)	(0.125)	(0.092)
Age	7.673***	6.474***	4.832***	7.384***	7.005***	4.508***
	(0.499)	(0.462)	(0.665)	(0.828)	(0.818)	(1.089)
Age^2	0.949***	0.952***	0.961***	0.949***	0.948***	0.968**
	(0.004)	(0.004)	(0.009)	(0.007)	(0.007)	(0.015)
Internet Access [t-1]*Age			1.342**			1.612*
			(0.200)			(0.434)
Internet Access [t-1]*Age^2			0.994			0.98
			(0.010)			(0.017)
N	44,104 (5,513 individuals over 8 years)					

* p<0.10, ** p<0.05, *** p<0.01. Model (1) controls for gender, ethnicity, region, urban; Model (2): (1) + education, parents' education, family income, income from work, weeks of unemployment, previously married, previously cohabited, number of children in the household; Model (3): (2) + Internet Access [t-1]*age + Internet Access [t-1]*age^2.

Table A7. Fixed effects regression models (NLSY97)

Y = being in a partnership (Ref: No)	(1)	(2)	(3)
Internet Access	**0.979**	**0.997**	**0.226*****
	**(0.132)**	**(0.135)**	**(0.074)**
Age	**12.516*****	**9.540*****	**8.092*****
	**(1.469)**	**(1.149)**	**(1.008)**
Age^2	**0.955*****	**0.966*****	**0.963*****
	**(0.008)**	**(0.008)**	**(0.008)**
Region (Ref: Northeast)			
North Central	1.539	1.542	1.595
	(0.765)	(0.806)	(0.849)
South	3.606***	3.272***	3.307***
	(1.572)	(1.469)	(1.514)
West	3.953***	3.684***	3.843***
	(1.931)	(1.839)	(1.952)
Urban Area	1.417**	1.411**	1.396**
	(0.207)	(0.210)	(0.209)
Enrolled In School		0.636***	0.635***
		(0.090)	(0.091)
Level of Education (Ref: < High School)		0.518	0.579
High School Diploma		(0.419)	(0.472)
		1.341	1.306
Some College		(1.328)	(1.301)
		1.979	2.068
College Degree or more		(1.819)	(1.911)
Log(Income from Job Past Year)		1.030*	1.031*
		(0.017)	(0.017)
Weeks Unemployed per Year		1	1.001
		(0.006)	(0.006)
Number of own children in household		2.311***	2.383***
		(0.353)	(0.358)
Internet Access*Age			**1.274*****
			**(0.062)**
N	22,320 (2,480 individuals for 9 years)		

* p<0.10, ** p<0.05, *** p<0.01

Table A8. Multinomial multilevel regression models (NLSY97) - Internet Access and Weekly Hours of TV Watched

**Y = being in a partnership (Ref: No)**	(1)	(2)	(3)
Internet Access	1.449*	0.093***	
	(0.277)	(0.064)	
Age	7.506***	5.490***	6.828***
	(1.448)	(1.121)	(1.393)
Age^2	0.969***	0.970***	0.972***
	(0.011)	(0.011)	(0.011)
TV Hours per week (Ref: Less than 2 hours per week)			
3 to 10 hours a week	1.562***	1.545**	0.597
	(0.265)	(0.263)	(0.395)
11 to 20 hours a week	2.387***	2.352***	1.919
	(0.492)	(0.486)	(1.513)
21 to 30 hours a week	2.188***	2.151***	1.466
	(0.613)	(0.602)	(1.579)
31 to 40 hours a week	2.963***	2.720**	36.420**
	(1.172)	(1.071)	(63.622)
More than 40 hours a week	1.375	1.325	8.963
	(0.511)	(0.491)	(12.629)
Internet Access * Age		1.368***	
		(0.103)	
TV Hours per week (Ref: Less than 2 hours per week) * Age			
3 to 10 hours a week * Age			1.116
			(0.081)
11 to 20 hours a week * Age			1.027
			(0.089)
21 to 30 hours a week * Age			1.043
			(0.122)
31 to 40 hours a week * Age			0.759
			(0.141)
More than 40 hours a week * Age			0.806
			(0.125)
N		22,005	

* p<0.10, ** p<0.05, *** p<0.01. Model (1): controls for gender, ethnicity, region, urban, education, parents' education, family income, income from work, weeks of unemployment, previously married, previously cohabited, number of children in the household + weekly hours of TV watched; Model (2): (1) + Internet Access*age; Model (3) controls for gender, ethnicity, region, urban, education, parents' education, family income, income from work, weeks of unemployment, previously married, previously cohabited, number of children in the household

Table A9. Multinomial multilevel regression models - Internet Access[t-1] (CPS)

Y = being in a partnership (Ref: No), OR	Different-sex Partnership (N=74,816)			Same-sex Partnership (N=1,340)		
	(1)	(2)	(3)	(1)	(2)	(3)
Internet Access [t-1]	1.944***	1.384***	0.205***	1.512***	1.341***	0.078***
	(0.032)	(0.025)	(0.022)	(0.102)	(0.096)	(0.033)
Age	1.308***	1.247***	1.193***	1.190***	1.144***	1.073***
	(0.003)	(0.003)	(0.004)	(0.010)	(0.011)	(0.014)
Age^2	0.998***	0.998***	0.999***	0.998***	0.999***	0.999***
	(0.000)	(0.000)	(0.000)	(0.000)	(0.000)	(0.000)
Age*Internet Access[t-1]			1.080***			1.122***
			(0.005)			(0.020)
Age^2*Internet Access[t-1]			0.999***			0.999***
			(0.000)			(0.000)
N	120,607	120,607	120,607	120,607	120,607	120,607

* p<0.10, ** p<0.05, *** p<0.01. All the specifications include dummies for US State and Year. Model (1) controls for gender, ethnicity, US state, metro area; Model (2): (1) + education, family income + weekly earnings + weeks of continuous unemployment + number children in the household; Model (3): (2) + Internet access * age + Internet access * age2

Table A10. Bivariate Probit Regression Models (CPS)

	Y = Being in a partnership (Ref: No), OR			Y = Internet Access (Ref: No Access), OR		
	(1)	(2)	(3)	(1)	(2)	(3)
Internet Access	1.341***	0.794***	0.690***	-	-	-
	(0.048)	(0.040)	(0.040)	-	-	-
Directly Use Computer at Work	-	-	-	1.909***	1.430***	1.431***
	-	-	-	(0.017)	(0.014)	(0.014)
Age	1.073***	0.997	0.995**	1.072***	1.024***	1.024***
	(0.002)	(0.002)	(0.002)	(0.002)	(0.002)	(0.002)
Age^2	0.999***	1.000***	1.000***	0.999***	1.000***	1.000***
	(0.000)	(0.000)	(0.000)	(0.000)	(0.000)	(0.000)
Age*Internet Access			1.003***		-	-
			(0.001)		-	-
N			104,151			

* p<0.10, ** p<0.05, *** p<0.01. All the specifications include dummies for US State and Year. Model (1) controls for gender, ethnicity, US state, metro area; Model (2): (1) + education, family income + weekly earnings + weeks of continuous unemployment + number children in the household; Model (3): (2) + Internet access * age.

Table A11. Descriptive Statistics - By Internet Access Information (CPS)

**(Weighted)**	**Internet Access Info NOT Missing**	**Internet Access Info MISSING**
Partnership Status (%)		
Not in a partnership	44.9	46.0
In a different-sex partnership	54.8	53.6
In a same-sex partnership	0.3	0.4
% Female	47.1	49.6
Mean Age	49.4	50.6
Race (%)		
White	71.0	68.0
Black	12.3	12.8
Hispanic	11.4	12.7
Asian	3.0	4.2
American Indian	0.7	0.6
Other/Mixed	1.7	1.7
Region (%)		
North East	18.5	17.9
North Central	22.7	22.2
South	36.8	37.7
West	22.0	22.2
% Not in metro area	17.0	15.3
Level of Education (%)		
Less than High School	13.6	11.0
High School	29.7	29.1
Some College	27.7	29.2
College Degree or more	29.0	30.8
Family Income (%)		
< $25,000	26.9	25.8
$25,000-49,999	25.4	26.2
$50,000-74,999	16.1	18.0
$75,000 and over	21.7	27.3
Missing	10.0	2.8
Avg. Earnings per Week (if working)	814.7	115.5
Avg. # Weeks Consecutive Unemployment (if >0)	28.8	33.6
Number of Children in the HH	0.76	0.74
N	619,158	333,734

Table A12. Partnership Status and Internet Access (NLSY97) - Logit models

Y = being in a partnership (Ref: No), OR	(1)	(2)	(3)
Internet Access	0.800**	0.966	0.109***
	(0.089)	(0.109)	(0.050)
Age	21.232***	15.235***	12.058***
	(1.785)	(1.195)	(1.677)
Age^2	0.916***	0.930***	0.932***
	(0.005)	(0.005)	(0.009)
Internet Access*Age			1.494***
			(0.220)
Internet Access*Age^2			0.992
			(0.011)
N	49,617 (5,513 individuals over 9 years)		

* p<0.10, ** p<0.05, *** p<0.01. Model (1) controls for gender, ethnicity, region, urban; Model (2): (1) + education, parents' education, family income, income from work, weeks of unemployment, number of children in the household; Model (3): (2) + Internet Access*age + Internet Access*age2.

Table A13. Partnership Status and Internet Access (CPS) - Logit Models

Y = being in a partnership (Ref: No), OR	(1)	(2)	(3)
Internet Access	2.493***	1.703***	1.357***
	(0.016)	(0.013)	(0.071)
Age	1.106***	1.053***	1.047***
	(0.001)	(0.001)	(0.002)
Age^2	0.999***	1.000***	1.000***
	(0.000)	(0.000)	(0.000)
Age*Internet Access			1.002
			(0.002)
Age^2*Internet Access			1.000**
			(0.000)
N	619158	619158	619158

* p<0.10, ** p<0.05, *** p<0.01. All the specifications include dummies for US State and Year. Model (1) controls for gender, ethnicity, US state, metro area; Model (2): (1) + education, family income + weekly earnings + weeks of continuous unemployment + number children in the household; Model (3): (2) + Internet access * age + Internet access * age squared.

Figure A1. Predicted probability of being in a partnership by internet access and age, by year (CPS)

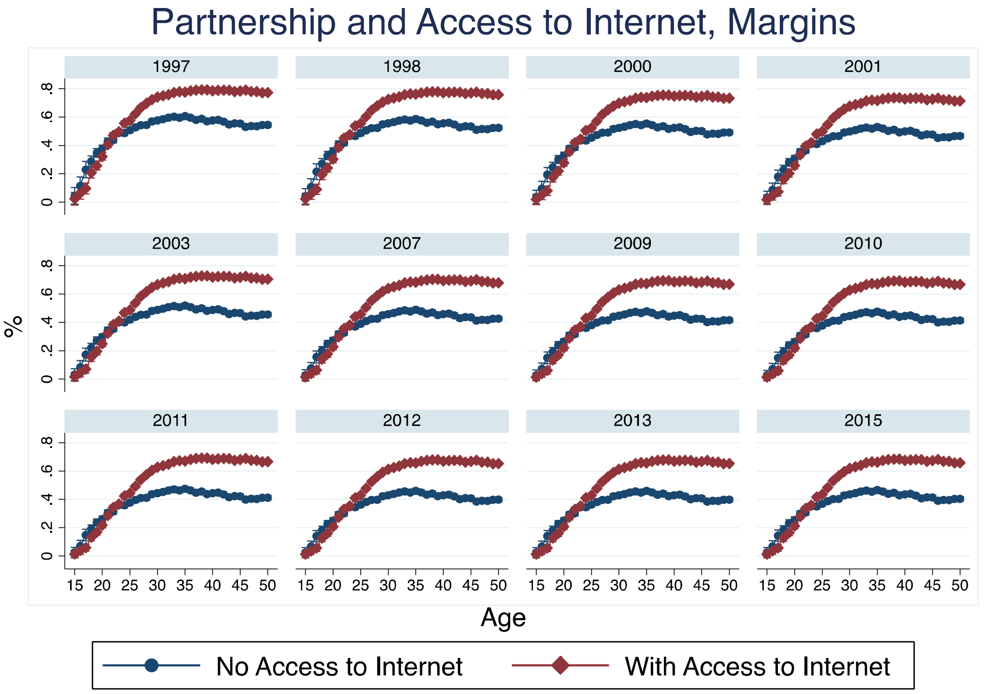

Source: CPS Data

Figure A2. Predicted probability of being in a partnership by Internet access and age, by year – with controls included (CPS)

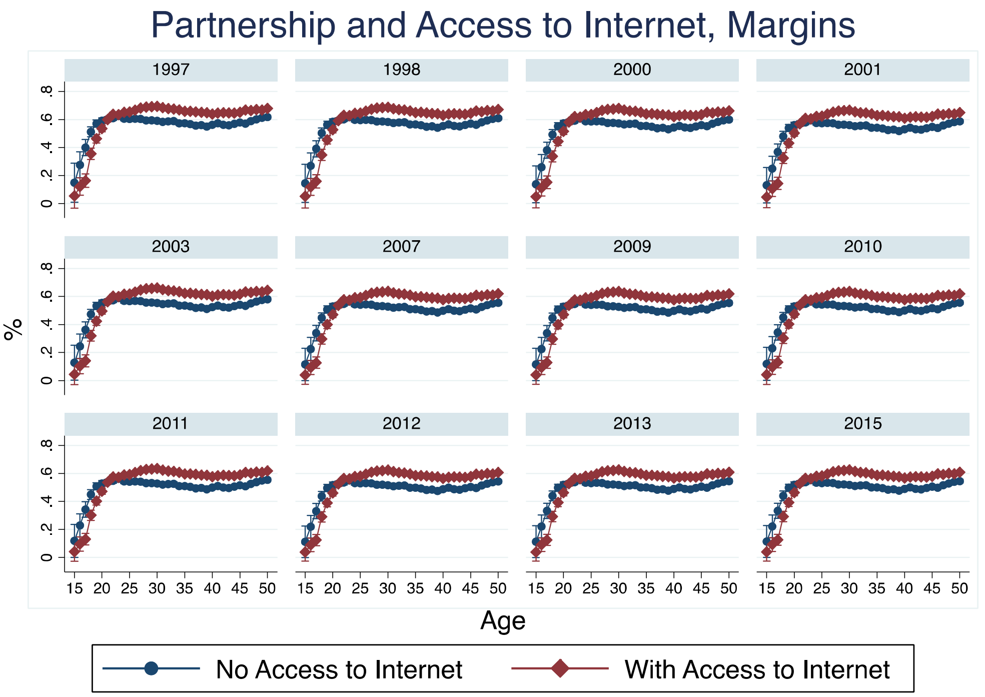

Source: CPS Data

Figure A3. Predicted probability of being in a partnership by Internet access and gender – including controls (CPS)

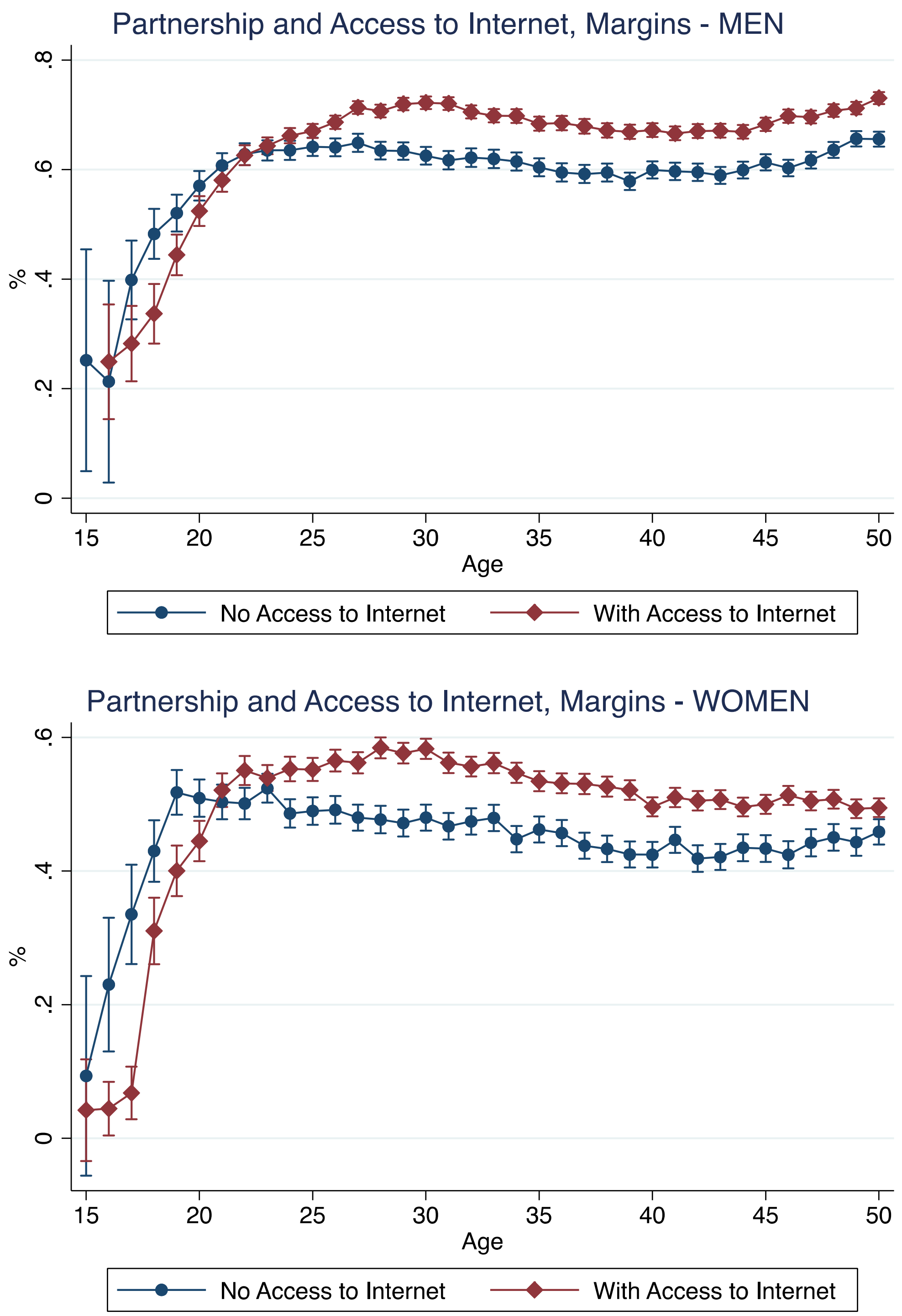

Figure A4. Predicted probability of being in a partnership by Internet access, All Locations - with confounders (CPS)

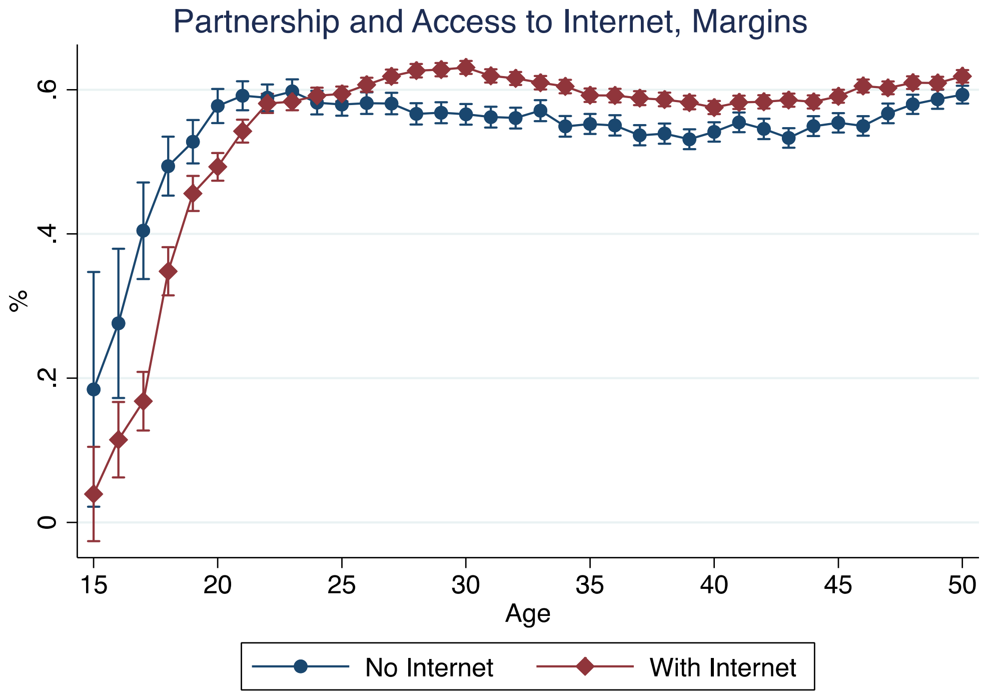

Supplement: Supplementary Material [file RPST_A_1999485_SM3483.pdf]
